# Research progress on the role of decorin in the development of oral mucosal carcinogenesis

**DOI:** 10.32604/or.2024.053119

**Published:** 2025-02-28

**Authors:** YONG RAO, XIAO CHEN, KAIYU LI, MINHAI NIE, XUQIAN LIU

**Affiliations:** 1 Department of Periodontics and Oral Mucosal Diseases, The Afliated Stomatology Hospital, Southwest Medical University, Luzhou, 646000, China; 2 Department of Basic Medicine of Stomatology, The Afliated Stomatology Hospital, Southwest Medical University, Luzhou, 646000, China; 3 Institute of Stomatology, Southwest Medical University, Luzhou, 646000, China; 4 Luzhou Key Laboratory of Oral and Maxillofacial Reconstruction and Regeneration, Luzhou, 646000, China; 5 Department of Oral Medical Technology, Sichuan College of Traditional Chinese Medicine, Mianyang, 621000, China; 6 Department of Orthodontics, Mianyang Stomatological Hospital, Mianyang, 621000, China

**Keywords:** Decorin (DCN), Oral potentially malignant disorders (OPMDs), Oral leukoplakia (OLK), Oral lichen planus (OLP), Oral submucous fibrosis, Oral erythroplakia (OEL), Oral squamous cell carcinoma (OSCC)

## Abstract

Decorin (DCN) is primarily found in the connective tissues of various parts of the body, including the lungs, kidneys, bone tissue, aorta, and tendons. It is an important component of the extracellular matrix (ECM) and belongs to the class I small leucine-rich proteoglycans family. DCN is increasingly attracting attention due to its significant role in tumors, fibrotic diseases, and the regulation of vascular formation. Moreover, its anti-tumor properties have positioned it as a promising biomarker in the fight against cancer. Numerous studies have confirmed that DCN can exert inhibitory effects in various solid tumors, particularly in oral squamous cell carcinoma (OSCC), by activating its downstream pathways through binding with the epidermal growth factor receptor (EGFR) and mesenchymal-epithelial transition (MET) receptor, or by stabilizing and enhancing the expression of the tumor suppressor gene p53 to mediate apoptosis in cancer cells that have undergone mutation. The occurrence of OSCC is a continuous and dynamic process, encompassing the transition from normal mucosa to oral potentially malignant disorders (OPMDs), and further progressing from OPMDs to the malignant transformation into OSCC. We have found that DCN may exhibit a bidirectional effect in the progression of oral mucosal carcinogenesis, showing a trend of initial elevation followed by a decline, which decreases with the differentiation of OSCC. In OPMDs, DCN exhibits high expression and may be associated with malignant transformation, possibly linked to the increased expression of P53 in OPMDs. In OSCC, the expression of DCN is reduced, which can impact OSCC angiogenesis, and inhibit tumor cell proliferation, migration, and invasion capabilities, serving as a potential marker for predicting adverse prognosis in OSCC patients. This article reviews the current research status of DCN, covering its molecular structure, properties, and involvement in the onset and progression of oral mucosal carcinogenesis. It elucidates DCN’s role in this process and aims to offer insights for future investigations into its mechanism of action in oral mucosal carcinogenesis and its potential application in the early diagnosis and treatment of OSCC.

## Introduction

Oral potentially malignant disorders (OPMDs), as a class of clinical states or lesions with increased risk of malignant transformation [1], mainly include oral leukoplakia (OLK), oral lichen planus (OLP), oral submucous fibrosis (OSF), and oral erythroplakia (OEL) [2], etc., and its overall carcinogenicity rate is roughly 7.9% [1]. OLK, as one of the most common OPMDs, has an annual incidence of 2.6% [3] and an overall malignant rate of 9.5% [2]. Clinically, OLK can be categorized into homogeneous and non-homogeneous types, with non-homogeneous types presenting a higher risk of carcinoma compared to homogeneous types, with carcinoma rates of 14.5% and 3%, respectively [4]. In contrast, proliferative verrucous leukoplakia (PVL) has a higher cancer rate than OLK and other OPMDs, and most patients with PVL will eventually develop oral squamous cell carcinoma (OSCC), with a rate of about 49.5% [2].

Other OPMD types exhibit varying carcinogenicity rates, with rates of 1.4% for oral lichen planus (OLP), 5.2% for oral submucous fibrosis (OSF), and 33.1% for oral erythroplakia (OEL) [2]. OPMDs are often regarded as a critical state in the carcinogenic process of the oral mucosa, and interventions at this stage are important for stopping the progression of the disease and preventing OSCC.

Oral mucosal carcinogenesis is a dynamic process that progresses from normal oral mucosa to OPMDs, and further malignant transformation to OSCC. During the OPMDs stage, cells and tissues have not yet undergone carcinogenesis. Intervention at this stage can effectively halt the progression of the disease and achieve favorable treatment outcomes. Failure to intervene in OPMDs, and prolonged exposure to factors that promote the occurrence and development of OSCC, such as smoking [5], alcohol consumption [6], betel nut chewing [7,8], and HPV infection [9], will accelerate the transformation from OPMDs to OSCC.

The etiology of OSCC is often attributed to sustained exposure to pathogenic risk factors, leading to genetic alterations and dysregulation of the tumor microenvironment, ultimately resulting in the occurrence and progression of OPMDs to OSCC. Genetic alterations result in aberrant activation of oncogenic pathways, including epidermal growth factor receptor (EGFR) [10], Wnt β-catenin [11], PI3K/AKT/mTOR [12], mesenchymal-epithelial transition (Met) [13], and RAS/RAF/MAPK [14]. Additionally, disruption of inhibitory pathways like TP53/RB [15] significantly contributes to OSCC progression. Moreover, factors such as immunosuppression, stromal alterations, hypoxia, and imbalance of the oral microbiota may contribute to the dysregulation of the tumor microenvironment, thereby promoting OSCC progression [16–18].

OSCC is known for its early metastasis, invasion, and destruction of adjacent tissues and organs, resulting in low survival rates and high deformity rates [19]. This significantly impacts patient survival and quality of life. Early detection of OSCC significantly enhances prognosis and crucially increases survival rates. However, most early-stage OSCC cases are asymptomatic, leading to delayed detection and medical attention [20]. The lack of clinical diagnostic markers for OSCC often results in late diagnoses. Therefore, there is an urgent need for new biomarkers to enhance the diagnosis, treatment, and prognosis prediction for OSCC patients.

Decorin (DCN) is mainly distributed in the connective tissues of the lungs, kidneys, bone tissues, aorta, tendons, and other parts of the organisms. It is an important component of the extracellular matrix (ECM) and belongs to the family of class I small leucine-rich proteoglycans (SLRPs) [21]. DCN, as an inhibitor targeting multiple receptor tyrosine kinase (RTK), has received increasing attention for its important role in tumors, fibrotic diseases, and regulation of angiogenesis. It is considered a promising anti-tumor biomarker due to its anti-tumor properties, particularly for those biomarkers heavily reliant on RTK signaling. Numerous studies have demonstrated that DCN has inhibitory effects in many solid tumors [22–25]. It achieves this either by binding to EGFR and MET receptors, activating subsequent pathways, or by stabilizing and increasing the expression of the oncogene p53, thereby mediating apoptosis of mutated cancer cells. However, the roles and mechanisms of DCN in different microenvironments vary.

Defining the role and possible mechanisms of DCN in the continuous and dynamic process of oral mucosal carcinogenesis is crucial. This understanding is significant for early diagnosis and treatment of OSCC, and for in-depth research into the mechanism of oral mucosal carcinogenesis. Hence, this paper summarizes the correlation between DCN and oral mucosal carcinogenesis.

## The Structure and Functions of DCN

The DCN gene is situated on chromosome 12 at the specific locus 12q21-q22. In mammals, DCN is a dimeric structure composed of a single macromolecular core protein covalently linked to a glycosaminoglycan (GAG) chain of sulphated chondroitin or dermatoglycosan. It has a molecular weight of around 65 kDa, with the core protein weighing approximately 42 kDa.

### The structure of DCN

**Figure 1:**
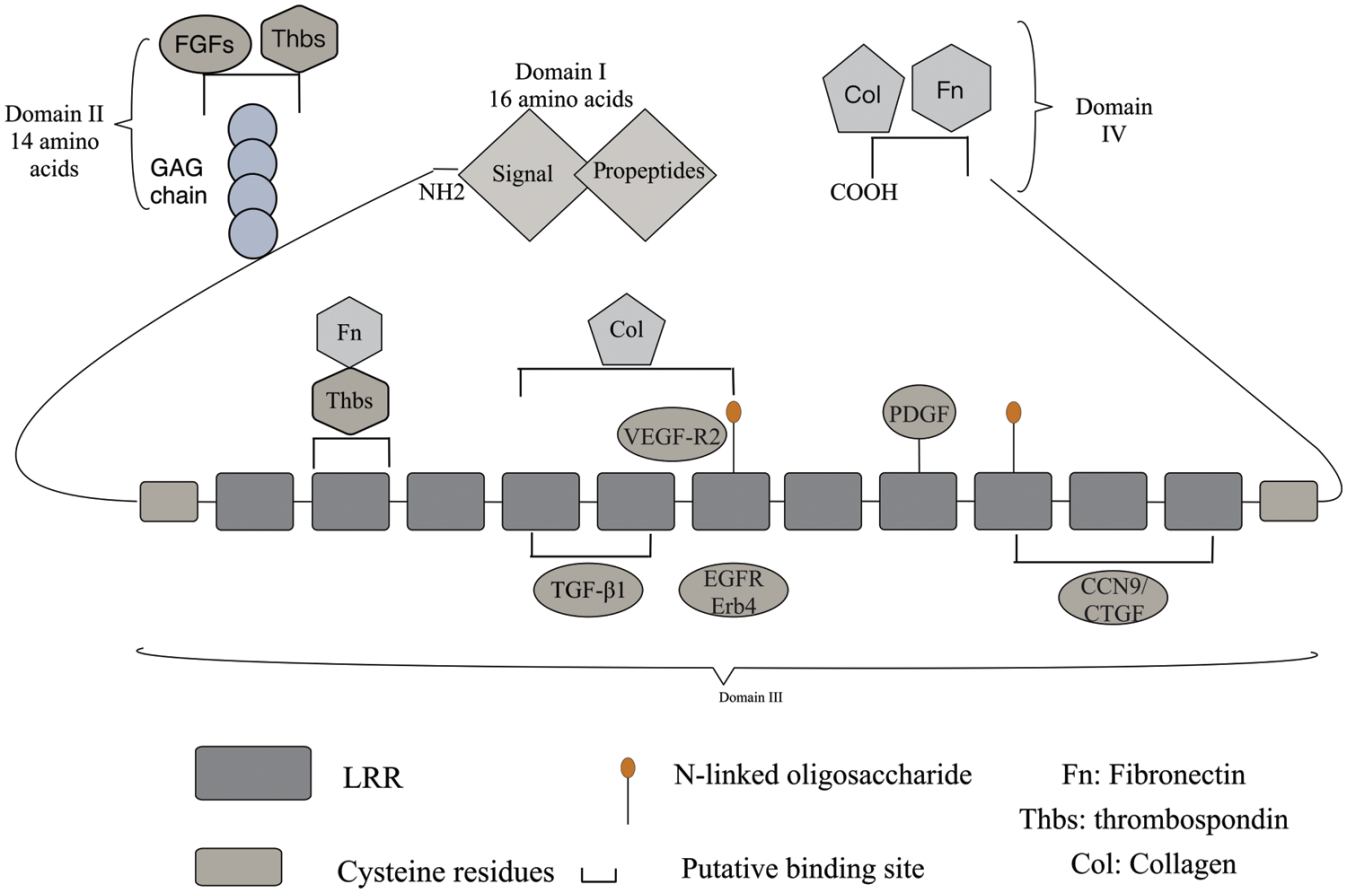
Structure of DCN: DCN consists of a core protein and a GAG side chain, which is linked to serine residues near the N-terminus. In domain I, the initial 16-amino acid-long signal peptide directs the core protein to the rough endoplasmic reticulum and is cleaved through a cotranslational pathway. In domain II, a 14-amino acid propeptide regulates the attachment of the GAG chain. The protein core is composed of LRRs rich in leucine residues, with cysteine-rich domains containing disulfide bonds on the sides. The central domain in domain III consists of 12 LRRs. Most DCN core protein ligands share an interface with the DCN core protein, which serves as a crucial hub for extracellular matrix, cell surface receptors, and growth factors. DCN exhibits high affinity binding sites for Col in LRRs 4-6 and low affinity sites at the C-terminus. The binding sites for TGF-β subtypes are LRR4 and LRR5 in domain II of the DCN core protein, while LRR7 domain interacts with EGFR and ErB4 to activate the MAPK pathway. DCN interacts with various growth factor signaling pathways. The carboxyl-terminal domains and binding sites for Fn and Col can be found in domain IV.

DCN contains a segment of leucine-rich repeat regions (LRRs). This segment belongs to the most characteristic family member of the SLRP family and includes four structural domains, Fig. 1.

Domain I: It is a signal peptide and prepeptide, and its main role is to guide immature macromolecular core proteins into the rough endoplasmic reticulum, to guide the GAG chain into the endoplasmic reticulum and to coordinate the binding of the GAG chain to the core proteins.

Domain II: It contains four cysteine residues and a mucopolysaccharide binding site, enabling specific binding to platelet reactive protein and fibroblast growth factor (FGF).

Domain III: The region most consistent with the properties of SLRP family members is the LRRs. This region is where DCN exerts its primary function and serves as the binding site for various signaling molecules such as transforming growth factor TGF-β1, connective tissue growth factor/CCN9 (CTGF), EGFR, collagen (Col), platelet-derived growth factor (PDGF), vascular endothelial growth factor receptor 2 (VEGF-R2), myostatin (MyoS), and fibronectin (FN) [21]. It interacts with different signaling molecules, leading to diverse biological effects.

Domain IV: Two cysteine residues form a cyclic carboxyl terminus, which serves as the binding site for fibronectin E to collagen fibers.

### The functions of DCN

DCN can elicit various effects by interacting with diverse signaling molecules, thereby contributing to a range of pathophysiological alterations. Numerous studies have demonstrated that DCN exhibits anticancer, antifibrotic, and angiogenesis-regulating properties, Table 1.

**Table 1:** Role and mechanism of DCN

Functions	Types of diseases	Model types	Mechanism	Manifestations
Cancer inhibition	Breast cancer	House mouse, cell	Inhibition of TGF-β1, β-catenin, c-Met and VEGF-α expression	Inhibits tumor proliferation, growth, metastasis and neoangiogenesis
	Cervical cancer	Cell		
	Colon cancer	House mouse, biopsy samples from patients	Stabilizes and increases the expression of the anticancer gene p53	Mediates apoptosis in mutated cancer cells
	Brain cancer, non-small cell lung cancer, cervical cancer, liver cancer.	Cell		
	Human umbilical vein endothelial cells (HUVEC)	Cell	Induction of autophagy in endothelial and tumor cells	Inhibition of tumor cell proliferation and growth and distant metastasis
Anti-fibrosis	Liver fibrosis	House mouse	Inhibition of TGF-β1 and TGF-β family activity	Inhibition of tissue and organ fibrosis
Regulation of angiogenesis	Breast cancer, cervical cancer	Cell	Activation of TSP-1 and TIMP-3; Inhibits MMP-9 and MMP-2; acting as a partial agonist of VEGFR2; promoting autophagic degradation of VEGF-A via adenylate-activated protein kinase (AMPK)	Inhibition of angiogenesis
	HUVEC	Cell		
	Primary placental microvascular endothelial cells	Cell		
	Keratitis, diabetic cardiomyopathy	House mouse	Up-regulation of VEGF expression through the IGF1R-AKT-VEGF signaling pathway; promoting integrin-collagen interactions	Promoting angiogenesis

### Cancer inhibitory effects of DCN

**Figure 2:**
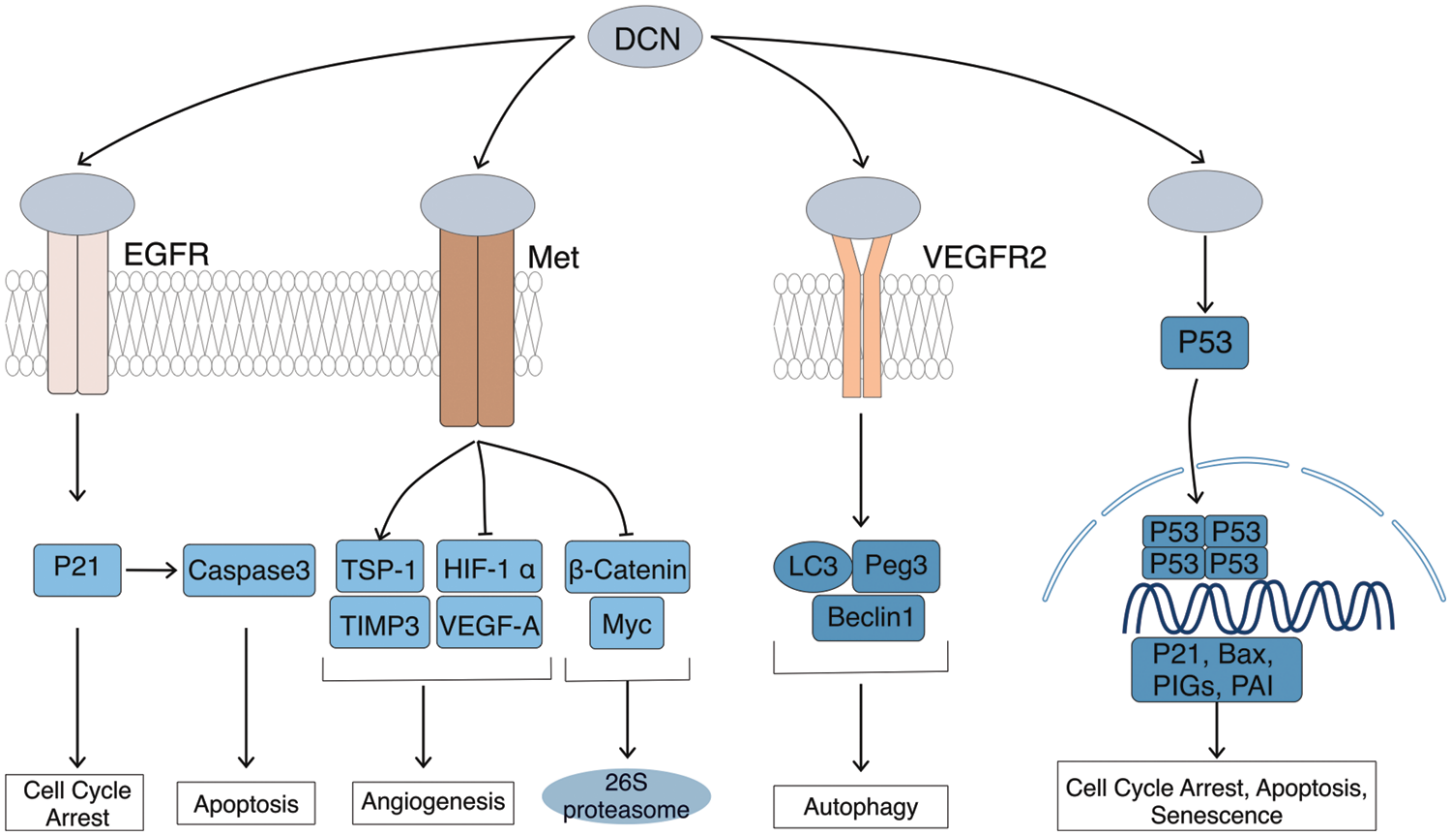
Cancer inhibitory effects of DCN. DCN induces EGFR phosphorylation, which triggers intracellular MAPK. This leads to enhanced expression of p21, resulting in cell cycle arrest and the release of Caspase 3, leading to cell apoptosis. Through Met receptor signaling, DCN downregulates β-catenin and MYC to inhibit tumor growth. It also suppresses angiogenesis by inhibiting HIF-1α and VEGFA. DCN-mediated VEGFR2 signaling inhibits angiogenesis and induces autophagy in endothelial cells by inhibiting mTOR and activating Beclin-1, LC3, and Peg3. Additionally, DCN stabilizes and increases p53 expression, thereby mediating cell cycle arrest and apoptosis.

Numerous studies have shown that the DCN gene acts as an anticancer gene. Its mechanisms of cancer inhibition include inhibiting tumor proliferation, growth, and neoangiogenesis by binding to cell-surface receptors like EGFR and Met, activating their pathways [26–29]. It also mediates apoptosis of mutated cancer cells by stabilizing and increasing the expression of the oncogene p53 [30–32]. Additionally, it inhibits tumor cell metastasis to distant sites by inducing autophagy of endothelial cells through VEGFR2 [33–36], Fig. 2. Horvath et al. [37] investigated the inhibitory effect of DCN on hepatocellular carcinoma cell lines, including HepG2, Hep3B, HuH7, and HLE, with varying molecular backgrounds and found that DCN exhibited a significant inhibitory effect on 3 out of the 4 hepatocellular carcinoma cell lines, each with different modes of action, regardless of the phenotypic and molecular characteristics of the carcinoma cell lines. Hu et al. [38] conducted a study on Inflammatory Breast Cancer (IBC) cells and discovered that the overexpression of DCN significantly reduced the engraftment, invasiveness, and tumor stem cell counts in IBC graft model mice. Additionally, it inhibited tumor growth and metastasis. In the research by Yu et al. [39], it was observed that the up-regulation of DCN expression led to the inhibition of lung metastasis in mice with triple-negative breast cancer (TNBC). Razie et al. [40] concluded that while DCN in urine and blood is not diagnostically valuable for prostate cancer (PC), in their study comparing DCN levels in urine and blood between PC and benign prostatic hyperplasia (BPH), it can serve as a prognostic marker for PC tumors when detected in tissues. The expression of DCN in hepatocellular carcinoma tissues was significantly lower than in normal tissues, suggesting that DCN may have inhibitory effects on the invasion and metastasis of cancer cells [41,42].

### Antifibrotic effects of DCN

**Figure 3:**
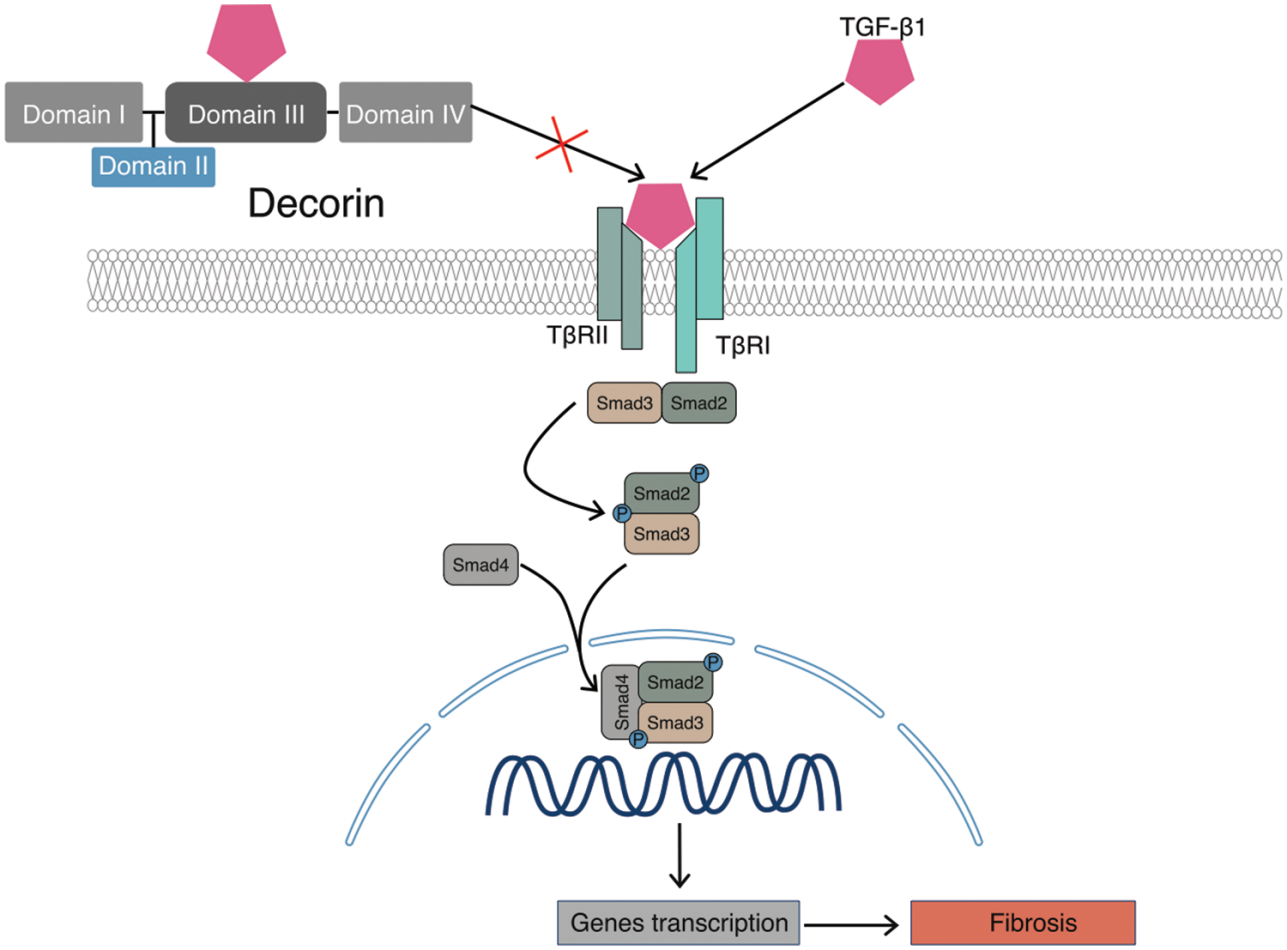
Antifibrotic effects of DCN. DCN binds to TGF-β1 through its domain III, thereby inhibiting its binding to receptors and consequently suppressing fibrosis in tissues and organs.

Numerous studies have demonstrated the anti-fibrotic effect of DCN. Fibrosis is characterized by an imbalance between the production and degradation of the extracellular matrix, resulting in its excessive accumulation. The primary anti-fibrotic mechanism of DCN involves binding of its structural domain III to TGF-β1, inhibiting the activity of this potent fibrogenic factor and thereby mitigating TGF-β1-induced tissue and organ fibrosis [43], Fig. 3. In addition to TGF-β1, DCN can also influence three other isoforms within the TGF-β family. In a therapeutic study by Chen et al. [44] on carbon tetrachloride-induced hepatic fibrosis in rats using lecithin-coupled decorin (PC-DCN) nanoliposomes, it was observed that the administration of these nanoliposomes significantly reduced the expression of hepatic function markers (ALT, AST, and TBIL) and effectively alleviated liver fibrosis. Li [45] concluded that DCN could inhibit or potentially treat hepatic fibrosis by reducing TGF-β1 levels in the hepatic tissues of Balb/c mice, thereby suppressing HSC activation, reducing ECM synthesis, and ultimately inhibiting or treating hepatic fibrosis induced by CCl4 in mice. A strong association exists between fibrosis and carcinogenesis, particularly in the pathological progression of OSF, an OPMD with the potential to transform malignantly into OSCC. The transformation mechanism may involve oxidative stress, epithelial-mesenchymal transition (EMT), acquisition of stem cell characteristics, and alterations in the Shh/Gli-1 axis [46,47].

### Bidirectional regulation of angiogenesis by DCN

Numerous studies have confirmed the significant role of blood vessels in the formation, growth, and metastasis of tumors [48–51]. Adequate vascular formation is essential for tumor growth. The absence of blood vessels can result in necrosis of the tumor tissue in that area. Conversely, regions with abundant blood supply are often sites of active tumor proliferation. Numerous researchers have demonstrated that the regulation of protein or gene expression impacting vascular formation influences tumor development [52–54].

**Figure 4:**
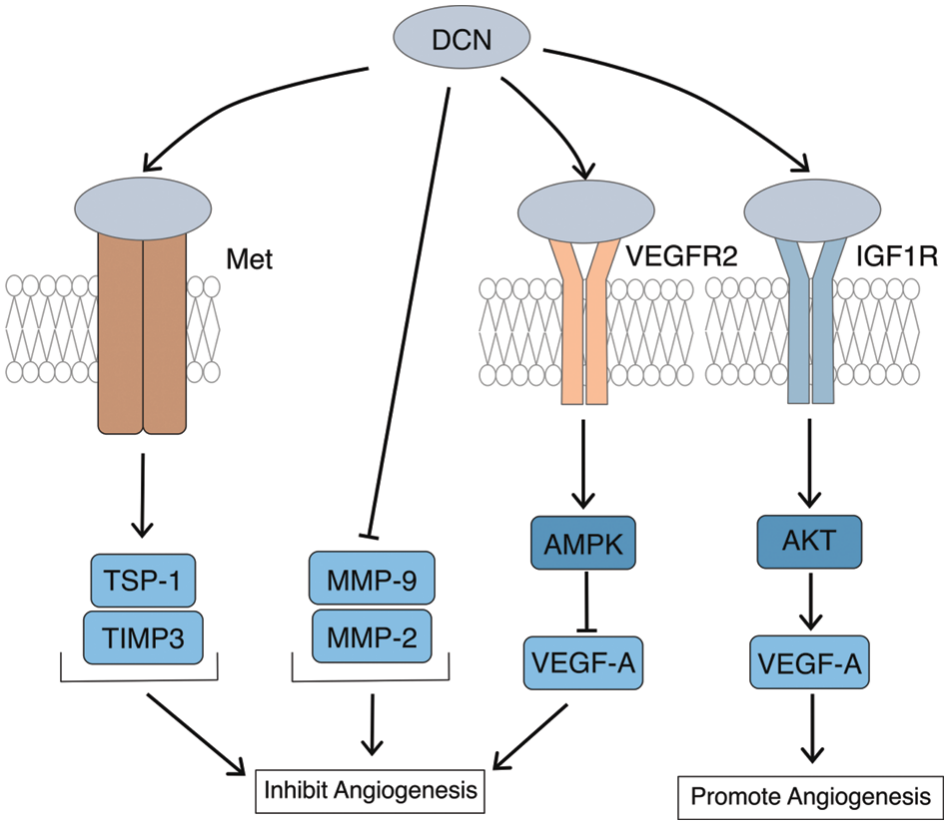
Bidirectional regulation of angiogenesis by DCN. DCN can activate TSP-1 and TIMP-3 through Met, while inhibiting the expression of MMP-9 and MMP-2 to suppress angiogenesis; DCN participates in and enhances the degradation of VEGF-A through VEGFR2, thus inhibiting angiogenesis; Overexpression of DCN can upregulate the expression of VEGF-A through the IGF1R-AKT-VEGF signaling pathway to promote vascular formation.

Studies have demonstrated that DCN exerts a bidirectional regulatory effect on angiogenesis [55]. Angiogenesis involves the participation of numerous protein molecules in the extracellular matrix. In the tumor microenvironment, exogenous DCN can activate the expression of angiogenesis inhibitors such as thrombospondin-1 (TSP-1) and tissue metalloproteinase tissue inhibitors of matrix metalloproteinase-3 (TIMP-3), while inhibiting pro-angiogenic substances like matrix metalloproteinase-9 (MMP-9) and matrix metalloproteinase-2 (MMP-2). During this process, DCN plays an inhibitory role in angiogenesis, Fig. 4. Neill et al. [56] demonstrated that DCN regulates the signal of VEGFR2, which inhibits angiogenesis by degrading potent vascular factors in the cytoplasm. In the study of angiogenesis in diabetic cardiomyopathy, Lai et al. [57] discovered that overexpression of DCN *in vitro* promotes angiogenesis by up-regulating VEGF expression through the IGF1R-AKT-VEGF signaling pathway. Conversely, outside the tumor microenvironment, DCN can promote angiogenesis. Evidence suggests that DCN promotes angiogenesis through enhancing integrin-collagen interaction [21]. In summary, DCN’s role in angiogenesis can be inhibitory or promotive, exhibiting bidirectionality mainly influenced by the cellular and molecular microenvironment of angiogenesis.

## The Role of DCN in the Process of Oral Mucosal Carcinogenesis

DCN is a crucial component of the extracellular matrix, playing a significant role in tumor development. DCN, functioning as a signaling molecule, can regulate cellular autophagy, thereby inhibiting tumor metastasis and proliferation [58]. Numerous studies have shown that DCN exerts an inhibitory effect on the development of various tumors [59–62].

### The role of DCNs in OPMDs

OPMDs represent a category of diseases with malignant potential, although their clinical manifestations and histopathological characteristics are not entirely identical. Research indicates that DCN expression was observed in 73.61% of OPMDs, 50.92% of OSCC, and 55.77% of healthy controls. Furthermore, in terms of distribution, DCN expression in the epithelial layer was found in 68.97% of OSF, 97.67% of OLK, and 5.56% of OSCC, while in the submucosal connective tissue, these percentages were 65.52%, 72.09%, and 56.48%, respectively [59].

### The role of DCNs in OLP

Currently, there is no direct evidence indicating the expression status or mechanism of action of DCN in OLP. However, a plethora of studies demonstrate that certain DCN-associated molecules play crucial roles in the pathogenesis and progression of OLP. For example, TGF-β1 plays a crucial role in suppressing the immune response to self-antigens, and the deficiency of TGF-β1 makes the body more susceptible to OLP [63,64]. The expression of P53 gradually increases, during the progression from normal mucosa to OLP, and further to OSCC, suggesting the potential involvement of P53 in the malignant transformation of OLP [65,66]. In OLP, the increase in angiogenesis is primarily attributed to the elevated expression of VEGF [67,68]. DCN, as an inhibitor of TGF-β1 and an activator of the VEGF pathway, may promote the occurrence and development of OLP by suppressing the expression of TGF-β1 and increasing VEGF expression to facilitate angiogenesis. However, this is merely speculative and requires further experimental validation.

### The role of DCNs in OLK

On one hand, Seema’s research demonstrates that in OLK tissues, the expression of DCN is significantly elevated compared to normal tissues. Furthermore, the expression of DCN is significantly correlated with VEGF-A expression and microvessel density, indicating a close association between DCN and vascular formation during the development of OLK [59]. On the other hand, P53 exhibits high expression in OLK. It is significantly associated with malignant transformation [69,70]. Additionally, the positive or high expression of Met also demonstrates a significant correlation with malignant transformation [69,71]. The high expression of P53 and Met, which should normally suppress the process of carcinogenesis, is paradoxically associated with the malignant transformation of OLK. DCN, as a molecule regulating the expression of P53 and Met, may counteract the inhibitory effects of P53 and Met on carcinogenesis by promoting tissue vascular formation, ultimately exhibiting a promoting effect in the progression of OLK.

### The role of DCNs in OSF

In the OSF organization, similar to OLK, DCN expression is significantly elevated in abnormal organizations and DCN is significantly correlated with VEGF-A expression and microvessel density [59]. Furthermore, Met positivity or high expression exhibits a significant correlation with malignant transformation [72], indicating an inhibitory effect on OSF progression by enhancing P53 expression [73]. We speculate that the reasons for this may be similar to those in OLK. However, unlike OLK, P53 does not exhibit a significant correlation with malignant transformation in OSF [73].

### The role of DCNs in OEL

Current research has not provided evidence directly linking DCN to the occurrence of OEL. However, parts of genes or proteins closely associated with DCN have shown a correlation with OEL. In OEL, the expression of P53 is elevated [74], while the expression of EGFR increases and further escalates as the condition progresses [75,76].

Researchers have conducted extensive studies on the role of DCN and its related molecules in OPMDs. The results suggest a potential dual role of DCN in OPMDs: On one hand, the heightened expression of DCN in OPMDs can promote angiogenesis in tissues by activating the VEGF pathway to enhance the expression of VEGF-A, thereby playing a facilitating role in the further progression of OPMDs. On the other hand, elevated levels of DCN can suppress the further development of OPMDs by promoting the expression of P53 and Met. Overall, a high expression of DCN may imply a poor prognosis in certain types of OPMDs, such as OLK, suggesting that DCN’s role in promoting the development of OPMDs outweighs its inhibitory effects.

Interestingly, the P53 gene shows high expression in various OPMDs and is significantly linked to malignant outcomes. However, P53, a tumor suppressor gene, has been extensively studied in the literature. Drugs targeting the P53 gene for treating malignant tumors, like APR-246 [77] and BOTI-2 [78], have entered clinical trials. The connection between these two aspects is paradoxical, and the molecular mechanisms are still not fully understood. This phenomenon, where DCN acts as a tumor suppressor gene but exhibits a potential promoting role in the malignant transformation of OPMDs, is reminiscent of the interaction between DCN and P53 that may facilitate the malignant transformation of OPMDs, indicating the need for further investigation.

Abnormal expression of DCN is also observed in the cancer progression of other sites such as the mammary duct. The expression of DCN decreases gradually during the development of mammary duct cancer, but it abnormally increases in the later stages [60,79]. Conversely, in the progression of cervical cancer, there is a trend of decreasing expression of DCN [80]. This differs from the expression pattern of DCN in the progression of oral mucosal carcinogenesis.

Some researchers’ findings are biased, for instance, P53 shows a significant association with malignant transformation in OLK, but not in OSF. This may be attributed to the fact that most researchers primarily utilize methods such as PCR, immunohistochemistry (IHC), and western blot (WB) to detect the expression of target proteins or mRNA. The outcomes are influenced by various factors, including sample size, sensitivity of experimental techniques, timing, and location of sampling, as well as the use of antibodies from different companies. The multitude of possible reasons and their inherent difficulty to fully control may lead to conflicting results among different researchers. Supplementing with additional testing methods or conducting detections at other molecular levels, such as co-immunoprecipitation and enzyme-linked immunosorbent assay (ELISA), could potentially enhance the accuracy of the results.

Many studies only focus on the detection of a single or a few specific molecules in OPMDs, and the results may not fully reflect reality. For example, molecules such as P53 and Met, known to inhibit the progression of cancer, have shown significant correlations with malignant transformation in OLK and OSF. Therefore, in future research, expanding the variety of molecules related to DCN may provide a more accurate reflection of the true role of DCN in OPMDs and the development of oral mucosal carcinogenesis.

This also suggests that further exploration is needed on the role of DCN in OPMDs, particularly the interaction between P53 and DCN, and the need for focused research in OPMDs with higher rates of malignant transformation such as OEL.

### The role of DCN in OSCC

Several studies have demonstrated decreased levels of DCN expression in OSCC tissues. Liu et al. [81] discovered that DCN expression in OSCC tissues was significantly lower than in normal tissues. Saleem et al. [82] discovered that the expression of DCN is also lower in squamous cell carcinoma of the tongue compared to normal tissues.

### The role of DCN in OSCC proliferation

Lai found that the expression of DCN protein in OSCC was significantly lower than in normal gingival tissues [83]. The level of DCN expression was negatively correlated with OSCC malignancy. As the tumor differentiation degree decreased, DCN expression gradually reduced. After upregulating the DCN gene expression, the levels of EGFR and C-Myc in human oral squamous carcinoma cells (HSC-3) decreased, while p21 expression increased. Guo confirmed this phenomenon through animal model experiments [84].

In OSCC, DCN may up-regulate p21 expression through the EGFR pathway. Additionally, through the Met pathway, DCN can bind to Met to form dimers, leading to the down-regulation of the oncogenic gene C-Myc. This down-regulation of C-Myc expression promotes p21 expression, which directly halts the cell cycle, thereby inhibiting tumor growth and metastasis [85,86]. These findings align with other scholars’ research on DCN’s role in inhibiting tumor formation [27,87].

DCN exerts its anticancer effects through various pathways. Further exploration is needed to understand how DCN inhibits tumor proliferation in OSCC, particularly through pathways involving TGF-β. The interaction between DCN and TGF-β strongly inhibits the proliferation of different cancer cell lines. This inhibition may be attributed to the binding of DCN to TGF-β1, leading to its segregation in the ECM and restricting its biological activity [88].

### The role of DCN in OSCC angiogenesis

Tumors require neovascularization to deliver micronutrients and oxygen to the growing tumor in order to progress to clinically significant sizes. Dil and other researchers demonstrated that the expression of DCN can significantly influence the angiogenic capacity in tumors [89]. Lowering DCN expression markedly inhibited the expression of IL-8, VEGF, and ANG-1. This inhibition may result from reduced IL-8 expression and the impact of ANG-1 on endothelial cell proliferation and migration. The findings of this study suggest a potential role of DCN in promoting angiogenesis in OSCC, which contradicts previous research. While numerous studies have confirmed the vasopressor effect of core proteoglycans in various types of tumors, including OSCC, and their down-regulation is linked to tumor vascularization levels in different cancer types [90–92]. Further investigation is required to validate the involvement of DCN in OSCC angiogenesis from multiple perspectives and to elucidate other angiogenic factors associated with DCN, such as TSP-1, TIMP-3, and AMPK.

### The role of DCN in OSCC invasion and metastasis

Dil and other researchers demonstrated that silencing DCN expression in OSCC led to decreased migration and invasion of OSCC [93]. This could be attributed to the downregulation of IL-8 expression by silencing DCN, which, as a growth factor secreted by OSCC, enhances MMP-7 expression, thereby inhibiting OSCC migration and proliferation. Nevertheless, the presence of DCN in OSCC lymph node metastasis has not been demonstrated, warranting further investigation.

In summary, DCN may inhibit the proliferation, invasion, and metastasis of tumor cells through pathways such as EFGR and Met during the developmental process of OSCC. However, as a bi-directional angiogenesis regulator, DCN has been shown to promote angiogenesis in OSCC, which plays a positive role in its development. DCN can inhibit tumor cell proliferation, invasion, and metastasis by upregulating the expression of IL-8 and MMP-7. Additionally, DCN can also positively impact OSCC invasion and metastasis through the promotion of IL-8 and MMP-7 expression. Thus, DCN can influence the progression of OSCC through both inhibitory and promotive mechanisms. Overall, its effect is inhibitory as evidenced in animal model experiments [84], although its role in humans remains unclear.

Current studies have shown that the antitumor activity of DCN includes several aspects. It promotes tumor cell autophagy to inhibit tumor progression [94], promotes endothelial cell autophagy to affect angiogenesis [33–36], and regulates immune and inflammatory responses [95–97]. However, these aspects are still missing in the current studies on the mechanism of DCN’s role in OSCC. The significance of tumor cell autophagy and immunity in tumorigenesis and development has garnered increasing attention, indicating a promising avenue for further research.

## DCN as a Biomarker of OSCC Prognosis

The treatment of OSCC primarily revolves around surgery, complemented by radiotherapy and/or chemotherapy. However, it is disheartening to note that such treatments have not significantly prolonged the survival of these patients [98,99]. Over the past two decades, the five-year survival rate has consistently hovered around approximately 60% [100]. One significant factor leading to poor prognosis in OSCC patients is the difficulty in early diagnosis. Early screening and timely therapeutic interventions can effectively halt the progression of OSCC, thereby increasing the survival rate of patients by 80% [101,102]. Early diagnosis of OSCC relies on clinical examination by physicians, however, some early symptoms of OSCC resemble oral ulcers or OPMDs, leading to potential confusion and resulting in misdiagnosis or underdiagnosis. The gold standard for diagnosing OSCC is pathological biopsy, yet the invasive nature of this procedure and variations in sampling sites may yield different pathological diagnoses. Therefore, there is an urgent need for methods with high specificity, operability, and non-invasive or minimally invasive techniques, such as detecting biomarkers in serum or saliva, to assist clinicians in screening for OSCC. Identifying reliable biomarkers is a crucial means for early diagnosis and prognostic prediction of OSCC. Currently, OSCC commonly includes the following types of biomarkers: IHC markers, such as PD-L1 [103,104], p16 [105,106], EGFR [107]; serological markers, including Carcinoma-specific carcinoembryonic antigen (CEA) [108], CA-125 [109,110], CYFRA 21-1 [111]; salivary markers, such as IL-1β, IFN-γ, TNF-α, IL-6, IL-8 [112,113].

Wu et al. [114] discovered that individuals with high plasma DCN content had a significantly reduced risk of esophageal squamous cell carcinoma (ESCC) compared to those with low plasma DCN content. This finding was observed in both early and late ESCC patients’ plasma. The reduced expression level of DCN serves as a reference for the early non-invasive detection of ESCC and suggests its potential as a biomarker for the early diagnosis and prognosis of OSCC.

Research by Seema and others shows that the low expression of DCN is significantly correlated with the overall survival rate of OSCC patients [59]. Kasamatsu and colleagues discovered that S-1 NAC chemotherapy sensitivity is high in OSCC patients with low DCN expression compared to those with high DCN expression, indicating a potential relationship between DCN and patient prognosis [115]. DCN may serve as a potential biomarker for early diagnosis and prognosis prediction in OSCC patients.

DCN is also linked to the prognosis of SCC in various locations. Ji and colleagues discovered that the Kaplan-Meier survival curve indicates a strong correlation between positive FHL1 and DCN expression and the prognosis of ESCC patients [116]. Furthermore, multivariate analysis revealed that the positive expression of FHL1 and DCN serves as an independent prognostic indicator for the overall survival of ESCC patients. Research by Biaoxue et al. demonstrates that the expression levels of DCN and p57 exhibit a positive correlation with the postoperative survival period of LSCC patients [117]. Additionally, low DCN expression is identified as a negative prognostic factor.

In conclusion, DCN holds the potential as a prognostic marker for OSCC and may serve as a minimally invasive early diagnostic marker in plasma. The downregulation of DCN may indicate an unfavorable prognosis for OSCC patients. If further research can establish the relationship between DCN expression in tissues, serum, or saliva and the prognosis of OSCC patients, it may lay the foundation for future clinical studies to predict the prognosis of OSCC patients through immunohistochemistry, serum, or saliva testing of DCN expression. This could offer new insights for early diagnosis and prognosis prediction of OSCC.

## Conclusion

Oral mucosal carcinogenesis progresses due to the complex interactions of genetic and environmental factors. Malignant cells interact continuously with the surrounding environment, with the extracellular matrix (ECM) playing a crucial role in this interaction. DCN, a significant ECM component, binds to various large molecular proteins to perform diverse functions. Upon binding to various receptors, DCN regulates crucial processes essential for tumor growth, invasion, and progression. These processes include the regulation of endothelial cell autophagy, tumor cell mitochondrial autophagy, cell cycle arrest, inflammation, and angiogenesis. DCN exerts tumor-suppressive effects by inhibiting tumor proliferation, regulating angiogenesis, and inducing inflammation and autophagy. It can be a promising target for tumor treatment and prognosis-related research.

In previous studies, it has been observed that the results from different researchers seem to vary, for instance, the association between EGFR regulated by DCN and the malignant transformation of OPMDs has shown different outcomes [118,119]. Various methods were employed in these studies to verify the expression of the target protein, including IHC and fluorescence in situ hybridization (FISH), which could be one of the reasons leading to disparate results. Researchers may opt for different experimental approaches, and the inconsistency in methods could impact the outcomes significantly. Therefore, selecting appropriate experimental methods is crucial for the accuracy of the results. For example, IHC is commonly used for the localization of target proteins and allows for semi-quantitative analysis; whereas WB enables a more precise semi-quantitative analysis of proteins; FISH can be utilized for the analysis of metaphase chromosomes and interphase cells, offering safety, speed, high sensitivity, and the ability to preserve probes for an extended period.

In the process of oral mucosal carcinogenesis, the expression of DCN shows a trend of initial increase followed by decrease, with an increase in DCN expression observed in OPMDs, while a decrease in expression is seen in OSCC, further decreasing with decreasing degree of differentiation. This pattern is inconsistent with the expression observed in the transformation process of squamous cell carcinoma in other sites [60,79,80].

In OPMDs, the expression of DCN is elevated. Among the genes associated with DCN, the expression of P53 and Met is increased, which is correlated with the malignant progression of OPMDs. Additionally, TGF-β1 is decreased, VEGF expression is increased, and angiogenesis is enhanced. DCN may have a dual effect in OPMDs. On one hand, high expression of DCN can activate the VEGF pathway, inhibit the TGF-β1 pathway, increase the expression of VEGF-A, promote the formation of blood vessels in tissues, thus facilitating the further development of OPMDs. The inhibition of TGF-β1 is also a contributing factor to the pathogenesis of some OPMDs. On the other hand, high expression of DCN may promote the expression of P53 and Met, inhibiting the further progression of OPMDs. Surprisingly, P53, known as a tumor suppressor gene, has been extensively documented in numerous studies. However, it exhibits high expression in various OPMDs and is significantly associated with malignant outcomes, presenting a contradictory relationship with its canonical role. The molecular mechanisms underlying this phenomenon remain unclear. The phenomenon is similar to DCN, which, as a tumor suppressor gene, may exhibit a potential promoting role in the malignant transformation of OPMDs. This implies that the interaction between DCN and P53 may promote the malignant transformation of OPMDs. Overall, in certain types of OPMDs, such as OLK, the high expression of DCN may suggest a poor prognosis, indicating that the promoting effect of DCN in the development of OPMDs may outweigh its inhibitory effect. In OPMDs, the high expression of DCN is more likely to be associated with a poor prognosis.

DCN can influence angiogenesis within OSCC, and its expression is closely associated with other biomarkers such as IL-8, VEGF, and ANG-1 in OSCC. DCN specifically manifests in promoting angiogenesis in OSCC. The occurrence and progression of OSCC are closely related to DCN, which may be associated with its proliferation, migration, and invasive abilities. The expression of DCN decreases with the degree of tumor differentiation. DCN inhibits the proliferation of OSCC through the EGFR and Met pathways and enhances the expression of IL-8 and MMP-7, thereby promoting the invasion and metastasis of OSCC. DCN can be utilized as a biomarker for the early diagnosis and prognosis of OSCC. Low DCN expression is an independent factor associated with mucosal cancer transformation and poor prognosis in patients with OSCC, Table 2.

**Table 2:** The role of DCN in the process of oral mucosal carcinogenesis

Stage	DCN expression	Relationship between DCN expression and disease	Possible mechanisms of action
OPMDs	Increased	Affects microvessel density; positively correlated with adverse prognosis	Promoting the expression of VEGF-A while inhibiting that of TGF-β1; enhancing the expression of the P53 and Met genes
OSCC	Decreases and decreases with the degree of differentiation	Negative correlation with malignancy; positive correlation with prognosis; no significant correlation with lymph node metastasis	Regulates the expression of EGFR, C-Myc and p21; correlates with IL-8, MMP-7, VEGF and ANG-1 expression

The findings suggest that DCN positively influences the advancement of oral mucosal carcinogenesis, serving as a potential biomarker and therapeutic target for the early detection and treatment of OSCC. Although significant advancements have been made in this research area, several issues remain unclear. For instance, does DCN contribute to the process of oral mucosal carcinogenesis by inducing endothelial cell autophagy and tumor cell autophagy? Does it promote or inhibit this process? Does its role vary at different stages of the carcinogenic process? Do the functions of tumor suppression, angiogenesis, cell autophagy, and others mediated by DCN act independently or synergistically? Further research is needed to comprehensively understand the role and mechanism of DCN in the progression of oral mucosal carcinogenesis.

In conclusion, DCN exhibits a bidirectional effect in the progression of oral mucosal carcinogenesis, showing an initial increase followed by a decrease, which decreases with the differentiation of OSCC. DCN is highly expressed in OPMDs and may be associated with malignant transformation. This may be related to the increased expression of P53 in OPMDs. Decreased expression of DCN in OSCC can impact angiogenesis, and inhibit tumor cell proliferation, migration, and invasion, making it a potential marker for predicting poor prognosis in OSCC patients.

## Data Availability

The data cited in the review article is available on the internet on various platforms.

## References

[1] Warnakulasuriya S, Kujan O, Aguirre-Urizar JM, Bagan JV, Gonzalez-Moles MA, Kerr AR, et al. Oral potentially malignant disorders: a consensus report from an international seminar on nomenclature and classification, convened by the WHO collaborating centre for oral cancer. Oral Dis. 2021;27(8):1862–80; 33128420 10.1111/odi.13704

[2] Iocca O, Sollecito TP, Alawi F, Weinstein GS, Newman JG, De Virgilio A, et al. Potentially malignant disorders of the oral cavity and oral dysplasia: a systematic review and meta-analysis of malignant transformation rate by subtype. Head Neck. 2020;42(3):539–55; 31803979 10.1002/hed.26006

[3] Zhang C, Li B, Zeng X, Hu X, Hua H. The global prevalence of oral leukoplakia: a systematic review and meta-analysis from 1996 to 2022. BMC Oral Health. 2023;23(1):645; 37670255 10.1186/s12903-023-03342-y PMC10481497

[4] Warnakulasuriya S, Ariyawardana A. Malignant transformation of oral leukoplakia: a systematic review of observational studies. J Oral Pathol Med. 2016;45(3):155–66; 26189354 10.1111/jop.12339

[5] Wang X, Xu J, Wang L, Liu C, Wang H. The role of cigarette smoking and alcohol consumption in the differentiation of oral squamous cell carcinoma for the males in China. J Cancer Res Ther. 2015;11(1):141–5; 25879352 10.4103/0973-1482.137981

[6] Ferraguti G, Terracina S, Petrella C, Greco A, Minni A, Lucarelli M, et al. Alcohol and head and neck cancer: updates on the role of oxidative stress, genetic, epigenetics, oral microbiota, antioxidants, and alkylating agents. Antioxidants. 2022;11(1):145; 35052649 10.3390/antiox11010145 PMC8773066

[7] Ko AM, Lee CH, Ko YC. Betel quid-associated cancer: prevention strategies and targeted treatment. Cancer Lett. 2020;477:60–9; 32112902 10.1016/j.canlet.2020.02.030

[8] Zhang P, Chua N, Dang S, Davis A, Chong KW, Prime SS, et al. Molecular mechanisms of malignant transformation of oral submucous fibrosis by different betel quid constituents-does fibroblast senescence play a role? Int J Mol Sci. 2022;23(3):1637; 35163557 10.3390/ijms23031637 PMC8836171

[9] Galati L, Chiocca S, Duca D, Tagliabue M, Simoens C, Gheit T, et al. HPV and head and neck cancers: towards early diagnosis and prevention. Tumour Virus Res. 2022;14:200245; 35973657 10.1016/j.tvr.2022.200245 PMC9420391

[10] Ribeiro FA, Noguti J, Oshima CT, Ribeiro DA. Effective targeting of the epidermal growth factor receptor (EGFR) for treating oral cancer: a promising approach. Anticancer Res. 2014;34(4):1547–52; 24692681

[11] Liu F, Millar SE. Wnt/beta-catenin signaling in oral tissue development and disease. J Dent Res. 2010;89(4):318–30. doi:10.1177/0022034510363373; 20200414 PMC3140915

[12] Su YC, Lee WC, Wang CC, Yeh SA, Chen WH, Chen PJ. Targeting PI3K/AKT/mTOR signaling pathway as a radiosensitization in head and neck squamous cell carcinomas. Int J Mol Sci. 2022;23(24):15749. doi:10.3390/ijms232415749; 36555391 PMC9778923

[13] Speight PM, Epstein J, Kujan O, Lingen MW, Nagao T, Ranganathan K, et al. Screening for oral cancer-a perspective from the global oral cancer forum. Oral Surg Oral Med Oral Pathol Oral Radiol. 2017;123(6):680–7. doi:10.1016/j.oooo.2016.08.021; 27727113

[14] Hsieh MJ, Ho HY, Lo YS, Lin CC, Chuang YC, Abomughaid MM, et al. Semilicoisoflavone B induces apoptosis of oral cancer cells by inducing ROS production and downregulating MAPK and Ras/Raf/MEK signaling. Int J Mol Sci. 2023;24(5):4505. doi:10.3390/ijms24054505; 36901935 PMC10003514

[15] Zhang J, Chen T, Yang X, Cheng H, Spath SS, Clavijo PE, et al. Attenuated TRAF3 fosters activation of alternative NF-κB and reduced expression of antiviral interferon, TP53, and RB to promote HPV-positive head and neck cancers. Cancer Res. 2018;78(16):4613–26; 29921694 10.1158/0008-5472.CAN-17-0642 PMC7983169

[16] Haas L, Elewaut A, Gerard CL, Umkehrer C, Leiendecker L, Pedersen M, et al. Acquired resistance to anti-MAPK targeted therapy confers an immune-evasive tumor microenvironment and cross-resistance to immunotherapy in melanoma. Nat Cancer. 2021;2(7):693–708. doi:10.1038/s43018-021-00221-9; 35121945 PMC7613740

[17] Teles F, Alawi F, Castilho RM, Wang Y. Association or causation? exploring the oral microbiome and cancer links. J Dent Res. 2020;99(13):1411–24. doi:10.1177/0022034520945242; 32811287 PMC7684840

[18] Elmusrati A, Wang J, Wang CY. Tumor microenvironment and immune evasion in head and neck squamous cell carcinoma. Int J Oral Sci. 2021;13(1):24. doi:10.1038/s41368-021-00131-7; 34341329 PMC8329257

[19] Ho AS, Kim S, Tighiouart M, Gudino C, Mita A, Scher KS, et al. Metastatic lymph node burden and survival in oral cavity cancer. J Clin Oncol. 2017;35(31):3601–9. doi:10.1200/JCO.2016.71.1176; 28880746 PMC5791830

[20] Hadzic S, Gojkov-Vukelic M, Pasic E, Dervisevic A. Importance of early detection of potentially malignant lesions in the prevention of oral cancer. Mater Sociomed. 2017;29(2):129–33. doi:10.5455/msm.2017.29.129-133; 28883777 PMC5544450

[21] Jarvinen TA, Prince S. Decorin: a growth factor antagonist for tumor growth inhibition. Biomed Res Int. 2015;2015:654765; 26697491 10.1155/2015/654765 PMC4677162

[22] Chen H, Wang Z, Yang N, Zhang J, Liang Z. Decorin inhibits proliferation and metastasis in human bladder cancer cells by upregulating p21. Medicine. 2022;101(26):e29760; 35777025 10.1097/MD.0000000000029760 PMC9239591

[23] Zhang L, Liu C, Gao H, Zhou C, Qin W, Wang J, et al. Study on the expression profile and role of decorin in the progression of pancreatic cancer. Aging. 2021;13(11):14989–98; 34021540 10.18632/aging.203060 PMC8221302

[24] Basak D, Jamal Z, Ghosh A, Mondal PK, Dey TP, Ghosh S, et al. Reciprocal interplay between asporin and decorin: implications in gastric cancer prognosis. PLoS One. 2021;16(8):e255915. 10.1371/journal.pone.0255915 PMC8357146 34379688

[25] Deng M, Xue Y, Xu L, Wang Q, Wei J, Ke X, et al. Chrysophanol exhibits inhibitory activities against colorectal cancer by targeting decorin. Cell Biochem Funct. 2020;38(1):47–57; 31710116 10.1002/cbf.3445

[26] Horvath Z, Kovalszky I, Fullar A, Kiss K, Schaff Z, Iozzo RV, et al. Decorin deficiency promotes hepatic carcinogenesis. Matrix Biol. 2014;35(Suppl. 1):194–205. doi:10.1016/j.matbio.2013.11.004; 24361483 PMC4039715

[27] Buraschi S, Pal N, Tyler-Rubinstein N, Owens RT, Neill T, Iozzo RV. Decorin antagonizes Met receptor activity and down-regulates beta-catenin and Myc levels. J Biol Chem. 2010;285(53):42075–85. doi:10.1074/jbc.M110.172841; 20974860 PMC3009933

[28] Goldoni S, Humphries A, Nystrom A, Sattar S, Owens RT, McQuillan DJ, et al. Decorin is a novel antagonistic ligand of the Met receptor. J Cell Biol. 2009;185(4):743–54. doi:10.1083/jcb.200901129; 19433454 PMC2711571

[29] Zhu JX, Goldoni S, Bix G, Owens RT, McQuillan DJ, Reed CC, et al. Decorin evokes protracted internalization and degradation of the epidermal growth factor receptor via caveolar endocytosis. J Biol Chem. 2005;280(37):32468–79. doi:10.1074/jbc.M503833200; 15994311

[30] Hu J, Cao J, Topatana W, Juengpanich S, Li S, Zhang B, et al. Targeting mutant p53 for cancer therapy: direct and indirect strategies. J Hematol Oncol. 2021;14(1):157. doi:10.1186/s13045-021-01169-0; 34583722 PMC8480024

[31] Reszegi A, Horvath Z, Karaszi K, Regos E, Postnikova V, Tatrai P, et al. The protective role of decorin in hepatic metastasis of colorectal carcinoma. Biomolecules. 2020;10(8):1199. doi:10.3390/biom10081199; 32824864 PMC7465536

[32] Yoon AR, Hong J, Yun CO. Adenovirus-mediated decorin expression induces cancer cell death through activation of p53 and mitochondrial apoptosis. Oncotarget. 2017;8(44):76666–85. doi:10.18632/oncotarget.20800; 29100340 PMC5652734

[33] Torres A, Gubbiotti MA, Iozzo RV. Decorin-inducible Peg3 Evokes Beclin 1-mediated autophagy and thrombospondin 1-mediated angiostasis. J Biol Chem. 2017;292(12):5055–69. doi:10.1074/jbc.M116.753632; 28174297 PMC5377817

[34] Neill T, Sharpe C, Owens RT, Iozzo RV. Decorin-evoked paternally expressed gene 3 (PEG3) is an upstream regulator of the transcription factor EB (TFEB) in endothelial cell autophagy. J Biol Chem. 2017;292(39):16211–20. doi:10.1074/jbc.M116.769950; 28798237 PMC5625051

[35] Goyal A, Neill T, Owens RT, Schaefer L, Iozzo RV. Decorin activates AMPK, an energy sensor kinase, to induce autophagy in endothelial cells. Matrix Biol. 2014;34(Suppl. 4):46–54. doi:10.1016/j.matbio.2013.12.011; 24472739 PMC4080419

[36] Buraschi S, Neill T, Goyal A, Poluzzi C, Smythies J, Owens RT, et al. Decorin causes autophagy in endothelial cells via Peg3. Proc Natl Acad Sci U S A. 2013;110(28):E2582–91. doi:10.1073/pnas.1305732110; 23798385 PMC3710796

[37] Horvath Z, Reszegi A, Szilak L, Danko T, Kovalszky I, Baghy K. Tumor-specific inhibitory action of decorin on different hepatoma cell lines. Cell Signal. 2019;62(5):109354. doi:10.1016/j.cellsig.2019.109354; 31271881

[38] Hu XD, Villodre ES, Larson R, Rahal OM, Wang XP, Gong Y, et al. Decorin-mediated suppression of tumorigenesis, invasion, and metastasis in inflammatory breast cancer. Commun Biol. 2021;4(1):72. doi:10.1038/s42003-020-01590-0; 33452400 PMC7811004

[39] Yu Q, Xin K, Miao Y, Li Z, Fu S, Hu S, et al. Anti-tumor responses to hypofractionated radiation in mice grafted with triple negative breast cancer is associated with decorin induction in peritumoral muscles. Acta Biochim Biophys Sin. 2018;50(11):1150–7. doi:10.1093/abbs/gmy094; 30124739

[40] Rezaie R, Falakian Z, Mazloomzadeh S, Ayati M, Morakabati A, Teimouri DM, et al. While urine and plasma decorin remain unchanged in prostate cancer, prostatic tissue decorin has a prognostic value. Iran Biomed J. 2020;24(4):229–35. doi:10.29252/ibj.24.4.229; 32306717 PMC7275814

[41] Zheng X, Wang P, Li L, Yu J, Yu C, Xu L, et al. Cancer-associated fibroblasts promote vascular invasion of hepatocellular carcinoma via downregulating decorin-integrin β1 signaling. Front Cell Dev Biol. 2021;9:678670. doi:10.3389/fcell.2021.678670; 34504839 PMC8421641

[42] Reszegi A, Horvath Z, Feher H, Wichmann B, Tatrai P, Kovalszky I, et al. Protective role of decorin in primary hepatocellular carcinoma. Front Oncol. 2020;10:645. doi:10.3389/fonc.2020.00645; 32477937 PMC7235294

[43] Hu HH, Chen DQ, Wang YN, Feng YL, Cao G, Vaziri ND, et al. New insights into TGF-β/Smad signaling in tissue fibrosis. Chem Biol Interact. 2018;292:76–83. doi:10.1016/j.cbi.2018.07.008; 30017632

[44] Chen G, Zhu Y, Liang X, Wang X, Yu W, Guo J, et al. The effect of lecithins coupled decorin nanoliposomes on treatment of carbon tetrachloride-induced liver fibrosis. Biomed Res Int. 2020;2020(6):8815904. doi:10.1155/2020/8815904; 33415158 PMC7752282

[45] Li C. Effect of decorin on hepatic TGF-β1 and α-SMA expression in mice with CCl_4-induced liver fibrosis. Qinghai University: China; 2016.

[46] Chatterjee R, Ghosh B, Mandal M, Nawn D, Banerjee S, Pal M, et al. Pathophysiological relationship between hypoxia associated oxidative stress, Epithelial-mesenchymal transition, stemness acquisition and alteration of Shh/Gli-1 axis during oral sub-mucous fibrosis and oral squamous cell carcinoma. Eur J Cell Biol. 2021;100(1):151146. doi:10.1016/j.ejcb.2020.151146; 33418093

[47] Siriwardena B, Jayawardena K, Senarath NH, Tilakaratne WM. An evaluation of clinical and histopathological aspects of patients with oral submucous fibrosis in the background of oral squamous cell carcinoma. Biomed Res Int. 2018;2018:4154165; 30402477 10.1155/2018/4154165 PMC6198553

[48] Teofilo CR, Ferreira JA, Batista AC, Fechini JF, Sousa FB, Lima MM, et al. Mast cells and blood vessels profile in oral carcinogenesis: an immunohistochemistry study. Asian Pac J Cancer Prev. 2020;21(4):1097–102; 32334476 10.31557/APJCP.2020.21.4.1097 PMC7445991

[49] Kabiraj A, Jaiswal R, Singh A, Gupta J, Singh A, Samadi FM. Immunohistochemical evaluation of tumor angiogenesis and the role of mast cells in oral squamous cell carcinoma. J Cancer Res Ther. 2018;14(3):495–502; 29893305 10.4103/0973-1482.163693

[50] Thiem D, Schneider S, Venkatraman NT, Kumar VV, Brieger J, Frerich B, et al. Semiquantifiable angiogenesis parameters in association with the malignant transformation of oral leukoplakia. J Oral Pathol Med. 2017;46(9):710–6; 28036153 10.1111/jop.12544

[51] Wadhwan V, Sharma P, Saxena C, Venkatesh A. Grading angiogenesis in oral squamous cell carcinoma: a histomorphometric study. Indian J Dent Res. 2015;26(1):26–30; 25961611 10.4103/0970-9290.156792

[52] Itashiki Y, Harada K, Takenawa T, Ferdous T, Ueyama Y, Mishima K. Antitumor effects of bevacizumab in combination with fluoropyrimidine drugs on human oral squamous cell carcinoma. Oncol Lett. 2021;22(4):730. doi:10.3892/ol.2021.12991; 34429770 PMC8371954

[53] Li J, Liu X, Zang S, Zhou J, Zhang F, Sun B, et al. Small extracellular vesicle-bound vascular endothelial growth factor secreted by carcinoma-associated fibroblasts promotes angiogenesis in a bevacizumab-resistant manner. Cancer Lett. 2020;492:71–83. doi:10.1016/j.canlet.2020.08.030; 32860852

[54] Lu CC, Tsai HC, Yang DY, Wang SW, Tsai MH, Hua CH, et al. The chemokine CCL4 stimulates angiopoietin-2 expression and angiogenesis via the MEK/ERK/STAT3 pathway in oral squamous cell carcinoma. Biomedicines. 2022;10(7):1612. doi:10.3390/biomedicines10071612; 35884919 PMC9313364

[55] Jarvelainen H, Sainio A, Wight TN. Pivotal role for decorin in angiogenesis. Matrix Biol. 2015;43:15–26. doi:10.1016/j.matbio.2015.01.023; 25661523 PMC4560244

[56] Neill T, Chen CG, Buraschi S, Iozzo RV. Catabolic degradation of endothelial VEGFA via autophagy. J Biol Chem. 2020;295(18):6064–79. doi:10.1074/jbc.RA120.012593; 32209654 PMC7196639

[57] Lai J, Chen F, Chen J, Ruan G, He M, Chen C, et al. Overexpression of decorin promoted angiogenesis in diabetic cardiomyopathy via IGF1R-AKT-VEGF signaling. Sci Rep. 2017;7(1):44473. doi:10.1038/srep44473; 28290552 PMC5349602

[58] Gao Y. Oral histopathology. China: People’s Health Publishing House; 2020. p. 194–5 (In Chinese).

[59] Nayak S, Goel MM, Bhatia V, Chandra S, Makker A, Kumar S, et al. Molecular and phenotypic expression of decorin as modulator of angiogenesis in human potentially malignant oral lesions and oral squamous cell carcinomas. Indian J Pathol Microbiol. 2013;56(3):204–10. doi:10.4103/0377-4929.120366; 24152495

[60] Hosoya T, Oda G, Nakagawa T, Onishi I, Hosoya T, Ishiguro M, et al. Plasma levels of decorin increased in patients during the progression of breast cancer. J Clin Med. 2021;10(23):5530. doi:10.3390/jcm10235530; 34884232 PMC8658155

[61] Xie C, Mondal DK, Ulas M, Neill T, Iozzo RV. Oncosuppressive roles of decorin through regulation of multiple receptors and diverse signaling pathways. Am J Physiol Cell Physiol. 2022;322(3):C554–66. doi:10.1152/ajpcell.00016.2022; 35171698 PMC8917911

[62] Aljagthmi WA, Alasmari MA, Daghestani MH, Al-Kharashi LA, Al-Mohanna FH, Aboussekhra A. Decorin (DCN) downregulation activates breast stromal fibroblasts and promotes their pro-carcinogenic effects through the IL-6/STAT3/AUF1 signaling. Cells. 2024;13(8):680. doi:10.3390/cells13080680; 38667295 PMC11049637

[63] Lavanya N, Jayanthi P, Rao UK, Ranganathan K. Oral lichen planus: an update on pathogenesis and treatment. J Oral Maxillofac Pathol. 2011;15(2):127–32; 22529568 10.4103/0973-029X.84474 PMC3329692

[64] Pinas L, Alkhraisat MH, Suarez-Fernandez R, Anitua E. Biomolecules in the treatment of lichen planus refractory to corticosteroid therapy: clinical and histopathological assessment. Ann Anat. 2018;216:159–63; 29301093 10.1016/j.aanat.2017.12.006

[65] Shiva A, Zamanian A, Arab S, Boloki M. Immunohistochemical study of p53 expression in patients with erosive and non-erosive oral lichen planus. J Dent. 2018;19(2):118–23. PMC5960731 29854885

[66] Safadi RA, Al JS, Hammad HM, Hamasha AA. Oral lichen planus shows higher expressions of tumor suppressor gene products of p53 and p21 compared to oral mucositis. An immunohistochemical study. Arch Oral Biol. 2010;55(6):454–61; 20427035 10.1016/j.archoralbio.2010.03.019

[67] Hazzaa HH, El SM, Abdelgawad N, Gouda OM, Kamal NM. Correlation of VEGF and MMP-2 levels in oral lichen planus: an *in vivo* immunohistochemical study. J Oral Biol Craniofac Res. 2020;10(4):747–52; 33101894 10.1016/j.jobcr.2020.10.009 PMC7578756

[68] Al-Hassiny A, Friedlander LT, Parachuru V, Seo B, Hussaini HM, Rich AM. Upregulation of angiogenesis in oral lichen planus. J Oral Pathol Med. 2018;47(2):173–8; 29172242 10.1111/jop.12665

[69] Zhang X, Kim KY, Zheng Z, Bazarsad S, Kim J. Nomogram for risk prediction of malignant transformation in oral leukoplakia patients using combined biomarkers. Oral Oncol. 2017;72:132–9; 28797449 10.1016/j.oraloncology.2017.07.015

[70] Ramos-Garcia P, Gonzalez-Moles MA, Warnakulasuriya S. Significance of p53 overexpression in the prediction of the malignant transformation risk of oral potentially malignant disorders: a systematic review and meta-analysis. Oral Oncol. 2022;126:105734; 35091134 10.1016/j.oraloncology.2022.105734

[71] Saintigny P, William WJ, Foy JP, Papadimitrakopoulou V, Lang W, Zhang L, et al. Met receptor tyrosine kinase and chemoprevention of oral cancer. J Natl Cancer Inst. 2018;110(3):250–7; 29617836 10.1093/jnci/djx186 PMC5946820

[72] Bazarsad S, Zhang X, Kim KY, Illeperuma R, Jayasinghe RD, Tilakaratne WM, et al. Identification of a combined biomarker for malignant transformation in oral submucous fibrosis. J Oral Pathol Med. 2017;46(6):431–8; 27497264 10.1111/jop.12483 PMC5516200

[73] Zheng L, Guan ZJ, Pan WT, Du TF, Zhai YJ, Guo J. Tanshinone suppresses arecoline-induced epithelial-mesenchymal transition in oral submucous fibrosis by epigenetically reactivating the p53 pathway. Oncol Res. 2018;26(3):483–94. doi:10.3727/096504017X14941825760362; 28550687 PMC7844836

[74] Rabinovich OF, Rabinovich IM, Babichenko II, Umarova KV, Bekmurzova LF. Precancers of the oral mucosa: clinic, diagnostics. Stomatology. 2024;103(2):5–11 (In Russian). doi:10.17116/stomat20241030215; 38741528

[75] Tarle M, Raguz M, Muller D, Luksic I. Nuclear epidermal growth factor receptor overexpression as a survival predictor in oral squamous cell carcinoma. Int J Mol Sci. 2023;24(6):5816. doi:10.3390/ijms24065816; 36982894 PMC10056291

[76] Tarle M, Muller D, Raguz M, Luksic I. Significance of nuclear EGFR and ABCG2 expression in malignant transformation of oral potentially malignant disorders. Head Neck. 2022;44(12):2668–77. doi:10.1002/hed.27174; 36325600

[77] Sallman DA, DeZern AE, Garcia-Manero G, Steensma DP, Roboz GJ, Sekeres MA, et al. Eprenetapopt (APR-246) and azacitidine in TP53-mutant myelodysplastic syndromes. J Clin Oncol. 2021;39(14):1584–94. doi:10.1200/JCO.20.02341; 33449813 PMC8099410

[78] Lindemann A, Patel AA, Silver NL, Tang L, Liu Z, Wang L, et al. A novel thiosemicarbazone derivative, exhibits antitumor activity in HNSCC through p53-dependent and -independent mechanisms. Clin Cancer Res. 2019;25(18):5650–62. doi:10.1158/1078-0432.CCR-19-0096; 31308060 PMC6759991

[79] Van Bockstal M, Lambein K, Van Gele M, De Vlieghere E, Limame R, Braems G, et al. Differential regulation of extracellular matrix protein expression in carcinoma-associated fibroblasts by TGF-β1 regulates cancer cell spreading but not adhesion. Oncoscience. 2014;1(10):634–48. doi:10.18632/oncoscience.87; 25593993 PMC4278277

[80] Aljawad MF, Faisal A, Alqanbar MF, Wilmarth PA, Hassan BQ. Tandem mass tag-based quantitative proteomic analysis of cervical cancer. Proteomics Clin Appl. 2023;17(1):e2100105. doi:10.1002/prca.202100105; 36029187

[81] Liu M, Wang W, Piao S, Shen Y, Li Z, Ding W, et al. Relationship of biglycan and decorin expression with clinicopathological features and prognosis in patients with oral squamous cell carcinoma. J Oral Pathol Med. 2023;52(1):20–8. doi:10.1111/jop.13381; 36308714

[82] Saleem S, Aleem I, Atiq A, Tariq S, Babar A, Bakar MA, et al. Expression of cornulin in tongue squamous cell carcinoma. Ecancermedicalscience. 2021;15:1197. doi:10.3332/ecancer.2021.1197; 33889206 PMC8043688

[83] Lai YX, Nie MH, Chen X, Liu XQ. Correlation between decorin and oral squamous cell carcinoma. J Oral Sci Res. 2020;36(5):437–42.

[84] Guo MY, Li KY, Nie MH, Liu XQ. Effect of up-regulation of Decorin on expression of EGFR, C-Myc and p21 in nude mice with oral squamous cell carcinoma. Shanghai J Stoma. 2023;32(1):40–6 (In Chinese). 36973842

[85] Georgakilas AG, Martin OA, Bonner WM. p21: a two-faced genome guardian. Trends Mol Med. 2017;23(4):310–9; 28279624 10.1016/j.molmed.2017.02.001

[86] Tran TQ, Lowman XH, Reid MA, Mendez-Dorantes C, Pan M, Yang Y, et al. Tumor-associated mutant p53 promotes cancer cell survival upon glutamine deprivation through p21 induction. Oncogene. 2017;36(14):1991–2001; 27721412 10.1038/onc.2016.360 PMC5383530

[87] Yeh E, Cunningham M, Arnold H, Chasse D, Monteith T, Ivaldi G, et al. A signalling pathway controlling c-Myc degradation that impacts oncogenic transformation of human cells. Nat Cell Biol. 2004;6(4):308–18; 15048125 10.1038/ncb1110

[88] Zhang W, Ge Y, Cheng Q, Zhang Q, Fang L, Zheng J. Decorin is a pivotal effector in the extracellular matrix and tumour microenvironment. Oncotarget. 2018;9(4):5480–91; 29435195 10.18632/oncotarget.23869 PMC5797066

[89] Dil N, Banerjee AG. Knockdown of aberrantly expressed nuclear localized decorin attenuates tumour angiogenesis related mediators in oral cancer progression model *in vitro*. Head Neck Oncol. 2012;4:11; 22507529 10.1186/1758-3284-4-11 PMC3370992

[90] Neill T, Jones HR, Crane-Smith Z, Owens RT, Schaefer L, Iozzo RV. Decorin induces rapid secretion of thrombospondin-1 in basal breast carcinoma cells via inhibition of Ras homolog gene family, member A/Rho-associated coiled-coil containing protein kinase 1. FEBS J. 2013;280(10):2353–68; 23350987 10.1111/febs.12148 PMC3648593

[91] Neill T, Schaefer L, Iozzo RV. Decorin: a guardian from the matrix. Am J Pathol. 2012;181(2):380–7; 22735579 10.1016/j.ajpath.2012.04.029 PMC3409438

[92] Neill T, Painter H, Buraschi S, Owens RT, Lisanti MP, Schaefer L, et al. Decorin antagonizes the angiogenic network: concurrent inhibition of Met, hypoxia inducible factor 1alpha, vascular endothelial growth factor A, and induction of thrombospondin-1 and TIMP3. J Biol Chem. 2012;287(8):5492–506; 22194599 10.1074/jbc.M111.283499 PMC3285326

[93] Dil N, Banerjee AG. A role for aberrantly expressed nuclear localized decorin in migration and invasion of dysplastic and malignant oral epithelial cells. Head Neck Oncol. 2011;3(1):44. doi:10.1186/1758-3284-3-44; 21958730 PMC3198745

[94] Neill T, Torres A, Buraschi S, Owens RT, Hoek JB, Baffa R, et al. Decorin induces mitophagy in breast carcinoma cells via peroxisome proliferator-activated receptor gamma coactivator-1α (PGC-1α) and mitostatin. J Biol Chem. 2014;289(8):4952–68. doi:10.1074/jbc.M113.512566; 24403067 PMC3931056

[95] Merline R, Moreth K, Beckmann J, Nastase MV, Zeng-Brouwers J, Tralhao JG, et al. Signaling by the matrix proteoglycan decorin controls inflammation and cancer through PDCD4 and MicroRNA-21. Sci Signal. 2011;4(199):ra75. doi:10.1126/scisignal.2001868; 22087031 PMC5029092

[96] Yasuda M, Schmid T, Rubsamen D, Colburn NH, Irie K, Murakami A. Downregulation of programmed cell death 4 by inflammatory conditions contributes to the generation of the tumor promoting microenvironment. Mol Carcinog. 2010;49(9):837–48. doi:10.1002/mc.20660; 20607724 PMC3472367

[97] Davis BN, Hilyard AC, Lagna G, Hata A. SMAD proteins control DROSHA-mediated microRNA maturation. Nature. 2008;454(7200):56–61. doi:10.1038/nature07086; 18548003 PMC2653422

[98] Koyfman SA, Ismaila N, Crook D, D’Cruz A, Rodriguez CP, Sher DJ, et al. Management of the neck in squamous cell carcinoma of the oral cavity and oropharynx: ASCO clinical practice guideline. J Clin Oncol. 2019;37(20):1753–74; 30811281 10.1200/JCO.18.01921 PMC7098829

[99] Machiels JP, Rene LC, Golusinski W, Grau C, Licitra L, Gregoire V. Squamous cell carcinoma of the oral cavity, larynx, oropharynx and hypopharynx: eHNS-ESMO-ESTRO clinical practice guidelines for diagnosis, treatment and follow-up. Ann Oncol. 2020;31(11):1462–75; 33239190 10.1016/j.annonc.2020.07.011

[100] Zanoni DK, Montero PH, Migliacci JC, Shah JP, Wong RJ, Ganly I, et al. Survival outcomes after treatment of cancer of the oral cavity (1985–2015). Oral Oncol. 2019;90:115–21; 30846169 10.1016/j.oraloncology.2019.02.001 PMC6417804

[101] Madhura MG, Rao RS, Patil S, Fageeh HN, Alhazmi A, Awan KH. Advanced diagnostic aids for oral cancer. Dis Mon. 2020;66(12):101034; 32594996 10.1016/j.disamonth.2020.101034

[102] Silverman SJ, Kerr AR, Epstein JB. Oral and pharyngeal cancer control and early detection. J Cancer Educ. 2010;25(3):279–81; 20204575 10.1007/s13187-010-0045-6 PMC2933804

[103] Lenouvel D, Gonzalez-Moles MA, Ruiz-Avila I, Chamorro-Santos C, Gonzalez-Ruiz L, Gonzalez-Ruiz I, et al. Clinicopathological and prognostic significance of PD-L1 in oral cancer: a preliminary retrospective immunohistochemistry study. Oral Dis. 2021;27(2):173–82; 32583572 10.1111/odi.13509

[104] Dave K, Ali A, Magalhaes M. Increased expression of PD-1 and PD-L1 in oral lesions progressing to oral squamous cell carcinoma: a pilot study. Sci Rep. 2020;10(1):9705; 32546692 10.1038/s41598-020-66257-6 PMC7297711

[105] Doll C, Steffen C, Beck-Broichsitter B, Richter M, Neumann K, Pohrt A, et al. The prognostic significance of p16 and its role as a surrogate marker for human papilloma virus in oral squamous cell carcinoma: an analysis of 281 cases. Anticancer Res. 2022;42(5):2405–13; 35489745 10.21873/anticanres.15719

[106] Tokuzen N, Nakashiro KI, Tojo S, Goda H, Kuribayashi N, Uchida D. Human papillomavirus-16 infection and p16 expression in oral squamous cell carcinoma. Oncol Lett. 2021;22(1):528; 34055093 10.3892/ol.2021.12789 PMC8138897

[107] Chen IH, Chang JT, Liao CT, Wang HM, Hsieh LL, Cheng AJ. Prognostic significance of EGFR and Her-2 in oral cavity cancer in betel quid prevalent area cancer prognosis. Br J Cancer. 2003;89(4):681–6; 12915878 10.1038/sj.bjc.6601171 PMC2376917

[108] Mauro C, Passerini R, Spaggiari L, Galetta D, Radice D, Lentati P, et al. New and old biomarkers in the differential diagnosis of lung cancer: pro-gastrin-releasing peptide in comparison with neuron-specific enolase, carcinoembryonic antigen, and CYFRA 21-1. Int J Biol Markers. 2019;34(2):163–7; 30994045 10.1177/1724600819834235

[109] Zhang CZ, Cheng XQ, Li JY, Zhang P, Yi P, Xu X, et al. Saliva in the diagnosis of diseases. Int J Oral Sci. 2016;8(3):133–7; 27585820 10.1038/ijos.2016.38 PMC5113094

[110] Roi A, Rusu LC, Roi CI, Luca RE, Boia S, Munteanu RI. A new approach for the diagnosis of systemic and oral diseases based on salivary biomolecules. Dis Markers. 2019;2019:8761860; 30906485 10.1155/2019/8761860 PMC6398069

[111] Rajkumar K, Ramya R, Nandhini G, Rajashree P, Ramesh KA, Nirmala AS. Salivary and serum level of CYFRA 21-1 in oral precancer and oral squamous cell carcinoma. Oral Dis. 2015;21(1):90–6; 24304502 10.1111/odi.12216

[112] Chu HW, Chang KP, Hsu CW, Chang IY, Liu HP, Chen YT, et al. Identification of salivary biomarkers for oral cancer detection with untargeted and targeted quantitative proteomics approaches. Mol Cell Proteomics. 2019;18(9):1796–806; 31253657 10.1074/mcp.RA119.001530 PMC6731081

[113] Chiamulera M, Zancan CB, Remor AP, Cordeiro MF, Gleber-Netto FO, Baptistella AR. Salivary cytokines as biomarkers of oral cancer: a systematic review and meta-analysis. BMC Cancer. 2021;21(1):205. doi:10.1186/s12885-021-07932-3; 33639868 PMC7912500

[114] Wu IC, Wu DC, Huang CC, Lin HS, Chen YK, Tsai HJ, et al. Plasma decorin predicts the presence of esophageal squamous cell carcinoma. Int J Cancer. 2010;127(9):2138–46. doi:10.1002/ijc.25239; 20143390

[115] Kasamatsu A, Uzawa K, Minakawa Y, Ishige S, Kasama H, Endo-Sakamoto Y, et al. Decorin in human oral cancer: a promising predictive biomarker of S-1 neoadjuvant chemosensitivity. Biochem Biophys Res Commun. 2015;457(1):71–6. doi:10.1016/j.bbrc.2014.12.093; 25550184

[116] Ji C, Liu H, Xiang M, Liu J, Yue F, Wang W, et al. Deregulation of decorin and FHL1 are associated with esophageal squamous cell carcinoma progression and poor prognosis. Int J Clin Exp Med. 2015;8(11):20965–70; 26885026 PMC4723871

[117] Rong BX, Cai XG, Liu H, Ma H, Yang SY, Zhang W, et al. Decreased expression of decorin and p57(KIP2) correlates with poor survival and lymphatic metastasis in lung cancer patients. Int J Biol Markers. 2011;26(1):9–21. doi:10.5301/JBM.2011.6372; 21360479

[118] Nankivell P, Williams H, McConkey C, Webster K, High A, MacLennan K, et al. Tetraspanins CD9 and CD151, epidermal growth factor receptor and cyclooxygenase-2 expression predict malignant progression in oral epithelial dysplasia. Br J Cancer. 2013;109(11):2864–74. doi:10.1038/bjc.2013.600; 24201754 PMC3844903

[119] Taoudi BM, Saintigny P, Thomas SM, El-Naggar AK, Papadimitrakopoulou V, Ren H, et al. Epidermal growth factor receptor expression and gene copy number in the risk of oral cancer. Cancer Prev Res. 2010;3(7):800–9. doi:10.1158/1940-6207.CAPR-09-0163; 20570883 PMC2900459

